# Cost-effectiveness analysis of bacterial vaginosis screening strategies in women attempting for natural conception

**DOI:** 10.1186/s12962-026-00798-x

**Published:** 2026-07-15

**Authors:** Dengyan Qi, Hanyue Zheng, Mengjie Zhao, Lei Ba, Bei Wang, Xuening Zhang, Xiang Hong

**Affiliations:** 1 Key Laboratory of Environmental Medicine and Engineering of Ministry of Education, Department of Epidemiology and Health Statistics, School of Public Health, Southeast University, Nanjing, Jiangsu 210009 China; 2 National Health Commission Key Laboratory of Contraceptives Vigilance and Fertility Surveillance, Nanjing, 210036 China; 3 Jiangsu Provincial Medical Key Laboratory of Fertility Protection and Health Technology Assessment, Nanjing, 210036 China; 4 Jiangsu Health Development Research Center, Nanjing, 210036 China

**Keywords:** Bacterial vaginosis screening, Cost-effectiveness analysis, Nugent scoring, BV Blue

## Abstract

**Objectives:**

Bacterial vaginosis (BV) is associated with reduced fecundability in women. To address the limited accessibility and diagnostic sensitivity of current preconception BV screening in China, this study evaluated the cost-effectiveness of BV Blue, an accessible, self-administered rapid test.

**Methods:**

A decision tree model was constructed from a societal perspective to compare five screening strategies: No screening, Amsel criteria, BV Blue testing alone, sequential BV Blue–Nugent testing, and Nugent scoring alone. Effectiveness was defined as the number of pregnancies per 100,000 women over one year. Cost-effectiveness was evaluated using incremental cost-effectiveness ratios (ICERs), referenced against a willingness-to-pay (WTP) threshold based on China’s per capita GDP. Sensitivity analyses were conducted to assess model robustness.

**Results:**

In a simulated cohort of 100,000 women, BV Blue testing yielded 842 additional pregnancies compared with no screening (ICER: CNY 2,827 per pregnancy), well below the WTP threshold of CNY 99,665. The sequential and Amsel strategies were absolutely dominated by BV Blue testing. Compared with BV Blue testing, Nugent strategy yielded 171 additional pregnancies (ICER: CNY 198,051 per pregnancy), remaining below the three-fold per capita GDP threshold (CNY 298,995). However, sensitivity analyses indicated that the cost-effectiveness of Nugent strategy was not robust, being highly sensitive to BV Blue testing’s diagnostic sensitivity and pregnancy rates. On the cost-effectiveness acceptability curve, BV Blue testing consistently demonstrated the highest probability of being the optimal strategy.

**Conclusion:**

In the preconception period, all screening strategies yielded more pregnancies than no screening. Both BV Blue testing and Nugent scoring were cost-effective, whereas the Amsel and sequential strategies were not. BV Blue testing is a promising screening tool for preconception BV management. These findings may inform the selection of diagnostic pathways in China’s national preconception screening program.

**Supplementary Information:**

The online version contains supplementary material available at https://doi.org/10.1186/s12962-026-00798-x.

## Introduction

Infertility represents a major global reproductive health challenge, imposing substantial medical, economic, and psychological burdens on individuals, families, and healthcare systems [1, 2]. The World Health Organization estimated that the lifetime prevalence of infertility reached 17.5% between 1990 and 2021 [3]. This corresponds to approximately one in six couples worldwide. In China, this burden has increased considerably, with the prevalence rising from 12% in 2007 to 18% in 2020 [4].

Bacterial vaginosis (BV) is the most common vaginal infection among sexually active women of reproductive age [5]. It is characterized by the decrease in vaginal lactobacilli and the overgrowth of anaerobic bacteria, particularly *Gardnerella vaginalis*, leading to vaginal microbiota imbalance [6]. Accumulating evidence suggests that BV is associated with female infertility. Our previous prospective cohort study revealed that *Gardnerella* abundance in the vaginal microbiota was significantly higher in non-pregnant women than in pregnant women (*P* = 0.0029) [1]. A meta-analysis of 15 studies from our team further showed that BV-positive status is associated with an increased risk of infertility [7]. These findings are supported by broader studies, including Bayoumi et al.’s meta-analysis of 62 studies (*n* = 111,977), which reported that BV was associated with a nearly threefold increased risk of infertility (OR = 2.81) [8]. Lokken et al. similarly found that persistent BV was associated with reduced fecundability among women planning pregnancy [9]. Furthermore, Salah et al. provided direct evidence that BV treatment significantly improved cumulative pregnancy rates in women with unexplained infertility [6].

Randomised controlled trials are not currently feasible, as withholding treatment from women identified as BV-positive would be ethically unacceptable. These consistent findings from prospective cohort studies and systematic reviews therefore provide the strongest available evidence for the benefits of preconception BV screening and treatment, forming the clinical rationale for this health economic evaluation.

China has integrated preconception BV screening into its national reproductive health policy framework. The National Free Pre-conception Check-up Project, implemented nationwide in 2013, incorporates BV screening into routine assessments for women planning pregnancy [10]. Current guidelines classify BV as a Category B risk, recommending that affected women defer conception until successful treatment [11]. Currently, the clinical diagnosis of BV primarily relies on the Amsel clinical criteria and the Nugent scoring system. The former requires the presence of at least three of the following four clinical features: clue cells, a positive amine test, vaginal pH > 4.5, and homogeneous vaginal discharge. The latter quantifies bacterial morphotypes on Gram-stained vaginal smears, with a score of ≥ 7 considered diagnostic for BV [12].

However, preconception BV screening in China currently faces two major challenges in practice. With regard to accessibility, although the national coverage rate has reached 93.5% [13], actual participation rates among women of reproductive age vary considerably across regions. In one district of Shanghai, only 34.9% of early-pregnancy women had undergone preconception examination [14]. A 2019 survey in Guannan County found an even lower rate of 15.33%. Contributing factors include time constraints, inconvenient transportation, and limited awareness of the programme [15]. With regard to diagnostic sensitivity, the Amsel criteria currently serve as the primary diagnostic method. While simple, low-cost, and suitable for primary care, the Amsel criteria is subject to subjective interpretation and have a diagnostic sensitivity of only 60–72% [12]. Consequently, current screening practices are prone to underdiagnosis, leaving many BV-infected women undetected and missing the window for timely intervention during the preconception period.

Sialidase activity is now recognised as a key clinical marker for the diagnosis of BV [16]. BV Blue is a rapid point-of-care test that detects sialidase activity, can be self-administered at home, and delivers results within 10 min [17, 18]. Previous studies have demonstrated good specificity and predictive value across diverse populations [17, 18], supporting its potential as an accessible and reliable screening option. However, the cost-effectiveness of BV Blue testing relative to current preconception screening strategies has not been systematically evaluated.

To address these issues in current screening practice, we constructed a decision tree model to evaluate the cost-effectiveness of five preconception BV screening strategies from a societal perspective. The findings are intended to inform the optimisation of preconception BV screening pathways in China.

## Methods

### Decision analysis model

**Fig. 1:**
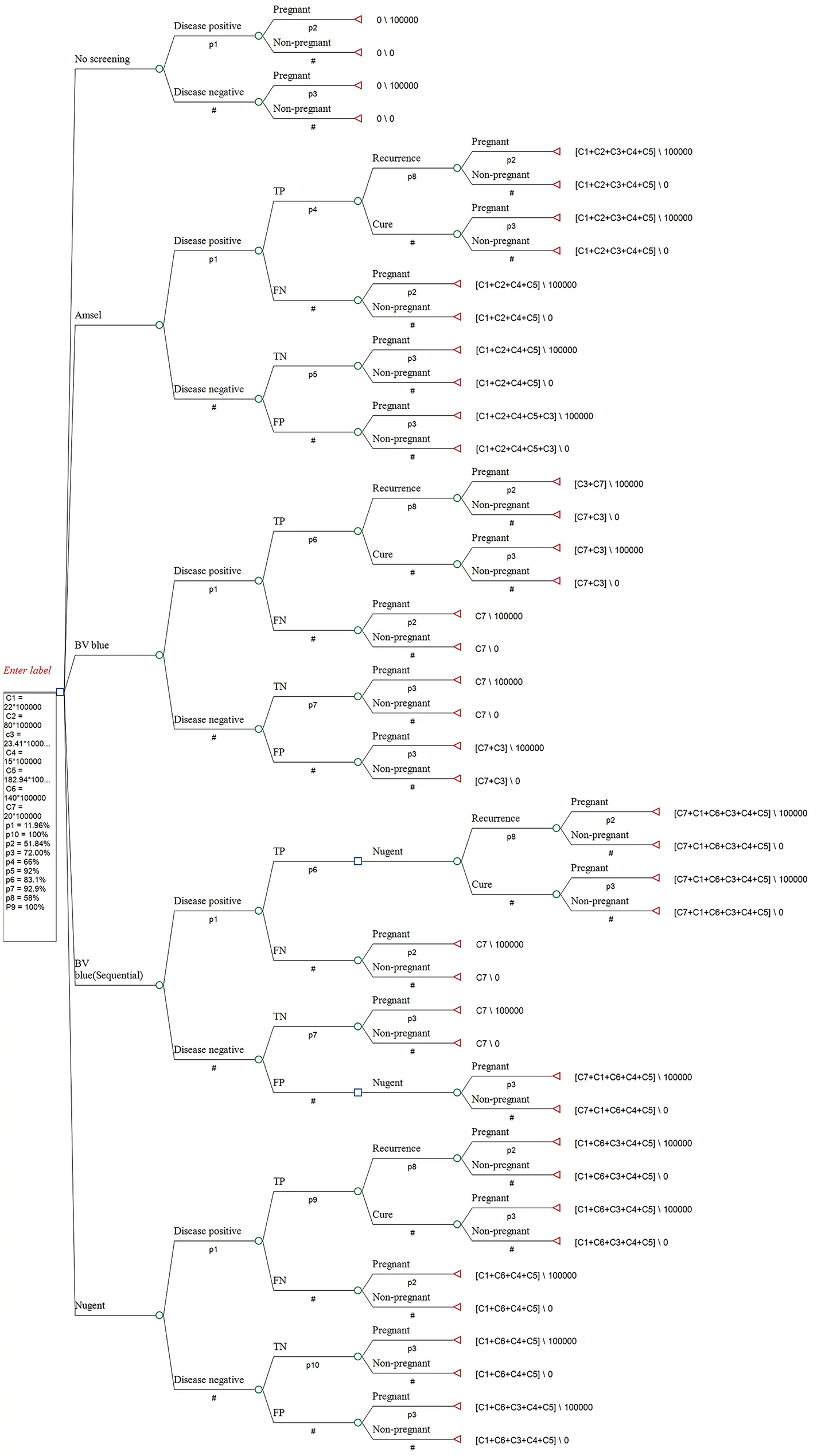
Decision tree model

A decision tree model (Fig. 1) was constructed using TreeAge Pro software from a societal perspective to evaluate the cost-effectiveness of five preconception BV screening strategies: (1) No screening (baseline); (2) Amsel-based clinical diagnosis; (3) BV Blue rapid testing alone; (4) Sequential BV Blue–Nugent strategy; and (5) Nugent scoring alone. The time horizon was set to one year, and discounting was not applied given the short analytic horizon.

In each strategy, women were stratified by true BV status (using Nugent scoring as the reference standard). They were then classified into true positive, false negative, true negative, and false positive groups based on the sensitivity and specificity of the respective screening method. Pregnant and non-pregnant outcomes were assigned based on BV status and treatment response. For treated women, cure and recurrence were modeled as two possible outcomes, with identical treatment regimens and cure rates applied across all strategies. In the sequential BV Blue–Nugent strategy, Nugent confirmation was performed only for BV Blue-positive cases to exclude false positives prior to treatment.

### Parameter input

#### Effectiveness

The primary effectiveness outcome was the expected number of pregnancies per 100,000 women over one year. The model first calculated the probability of pregnancy for an individual in each strategy by assigning an effectiveness value of 1 to “pregnant” outcomes and 0 to “non-pregnant” outcomes at the terminal nodes. The expected probability of pregnancy was then derived as the probability-weighted sum of these values. Finally, this individual-level probability was multiplied by 100,000 to represent the expected number of pregnancies in a hypothetical cohort of 100,000 women. Two BV-status-stratified pregnancy rates were incorporated: 51.84% for BV-positive women and 72.00% for BV-negative women (derivation detailed in Appendix A of the Supplementary Material).

#### Model probability parameters

The true prevalence of BV was set at 11.96%, based on a large-scale survey of 4,714 women in Beijing that used Nugent scoring as the diagnostic reference standard [19]. Accordingly, Nugent scoring was assigned a sensitivity and specificity of 100% as the reference method. The sensitivity and specificity of BV Blue testing were obtained from a meta-analysis conducted by our team (Appendix C of the Supplementary Material), while parameters for Amsel criteria and BV recurrence rates were sourced from existing literature. All probability parameters are summarized in Table 1.

**Table 1:** Model probability parameters

	Probability	Base-case Value	Range	Distribution	Source
P1	BV prevalence (Nugent-based)	11.96%	±20.00%	Beta	[19]
P2	Pregnancy rate (BV-positive)	51.84%	± 20.00%	Beta	[1, 7]
P3	Pregnancy rate (BV-negative)	72.00%	± 20.00%	Beta	[1, 7]
P4	Amsel’s sensitivity	66.00%	± 20.00%	Beta	[12]
P5	Amsel’s specificity	92.00%	± 20.00%	Beta	[12]
P6	BV Blue sensitivity	83.10%	± 20.00%	Beta	[17, 20–22]
P7	BV Blue Specificity	92.90%	± 20.00%	Beta	[17, 20–22]
P8	BV recurrence rate	58.00%	± 20.00%	Beta	[23]
P9	Nugent’s sensitivity	100.00%	80.00%-100.00%	Beta	-
P10	Nugent’s specificity	100.00%	80.00%-100.00%	Beta	-

#### Model costs

This study adopted a societal perspective, and costs were categorized into direct medical costs, direct non-medical costs, and indirect costs. Direct medical costs (screening and treatment) were derived from field surveys in Nanjing healthcare institutions and commercial pricing data. Direct non-medical costs included transportation expenses. Indirect costs, comprising productivity losses, were estimated using the human capital approach. Women who tested negative via home-based BV Blue self-testing incurred no transportation or productivity costs. Since Amsel-based screening and Nugent scoring are provided under the National Free Preconception Health Examination Programme, these costs were treated as government-borne resource consumption. All cost parameters and sensitivity analysis ranges are detailed in Table 2 and Appendix D of the Supplementary Material.

**Table 2:** Model cost parameters

	Cost item	Base-case value (CNY)	Range (CNY)	Distribution	Source
Amsel					
	Registration fee	22.00	±20.00%	Gamma	Healthcare institutions
	Amsel assessment	80.00	±20.00%	Gamma	Healthcare institutions
	Transportation fee	15.00	±20.00%	Gamma	Public transit pricing
	Treatment fee	23.41	±20.00%	Gamma	Healthcare institutions
	Productivity loss	182.84	±20.00%	Gamma	Nanjing Municipal Bureau of Statistics
BV Blue					
	BV Blue test kit	20.00	±20.00%	Gamma	Commercial platform
	Treatment fee	23.41	±20.00%	Gamma	Healthcare institutions
Sequential					
	Registration fee	22.00	±20.00%	Gamma	Healthcare institutions
	BV Blue test kit	20.00	±20.00%	Gamma	Commercial platform
	Gram stain (Nugent scoring)	140.00	±20.00%	Gamma	Healthcare institutions
	Transportation fee	15.00	±20.00%	Gamma	Public transit pricing
	Treatment fee	23.41	±20.00%	Gamma	Healthcare institutions
	Productivity loss	182.84	±20.00%	Gamma	Nanjing Municipal Bureau of Statistics
Nugent					
	Registration fee	22.00	±20.00%	Gamma	Healthcare institutions
	Gram stain(Nugent scoring)	140.00	±20.00%	Gamma	Healthcare institutions
	Transportation fee	15.00	±20.00%	Gamma	Public transit pricing
	Treatment fee	23.41	±20.00%	Gamma	Healthcare institutions
	Productivity loss	182.84	±20.00%	Gamma	Nanjing Municipal Bureau of Statistics

### Cost-effectiveness analysis and sensitivity analysis

We employed a cost-effectiveness analysis, using the incremental cost-effectiveness ratio (ICER) as the primary outcome measure. The willingness-to-pay (WTP) threshold was defined as the maximum amount society is willing to pay per unit of health gain (one additional pregnancy) [24]. Following WHO criteria [25], a strategy was considered highly cost-effective if the ICER was below 1× per capita GDP, cost-effective if between 1× and 3× per capita GDP, and not cost-effective if exceeding 3× per capita GDP. According to 2025 data from the National Bureau of Statistics [26], China’s per capita GDP was 99,665 CNY, establishing a WTP threshold range of 99,665–298,995 CNY per additional pregnancy.

One-way sensitivity analyses were performed on all model parameters by varying each value by ± 20%, in accordance with the Chinese Guidelines for Pharmacoeconomic Evaluations [27]. For probabilistic sensitivity analysis, a Monte Carlo simulation (1,000 iterations) was conducted. Cost parameters were assigned Gamma distributions, while transition probabilities and effectiveness parameters were assigned Beta distributions (standard deviation set at 20% of the mean). Results are presented as cost-effectiveness plane scatter plots and cost-effectiveness acceptability curves (CEACs).

## Results

### Base-case cost-effectiveness analysis

As shown in Table 3, the no screening strategy yielded 69,589 pregnancies over one year at zero cost.

**Table 3:** Cost-effectiveness analysis results of five preconception BV screening strategies (cohort of 100,000 women)

Strategy	Total Cost (CNY)	Incremental Cost (CNY)	Pregnancies (per 100,000)	Incremental Pregnancies	ICER	NMB(CNY)
No screening	0		69,589			6,935,574,131
BV blue	2,379,000	2,379,000	70,430	842	2,827	7,017,066,687
BV blue-Nugent	8,059,951	5,680,952	70,430	0	Dominated	7,011,385,734
Amsel	30,343,670	27,964,672	70,257	-173	Dominated	6,971,843,247
Nugent	36,273,984	33,894,985	70,602	171	198,051	7,000,228,612

Note: ICER, incremental cost-effectiveness ratio; NMB, net monetary benefit; NMB = pregnancies × WTP − total cost

**Fig. 2:**
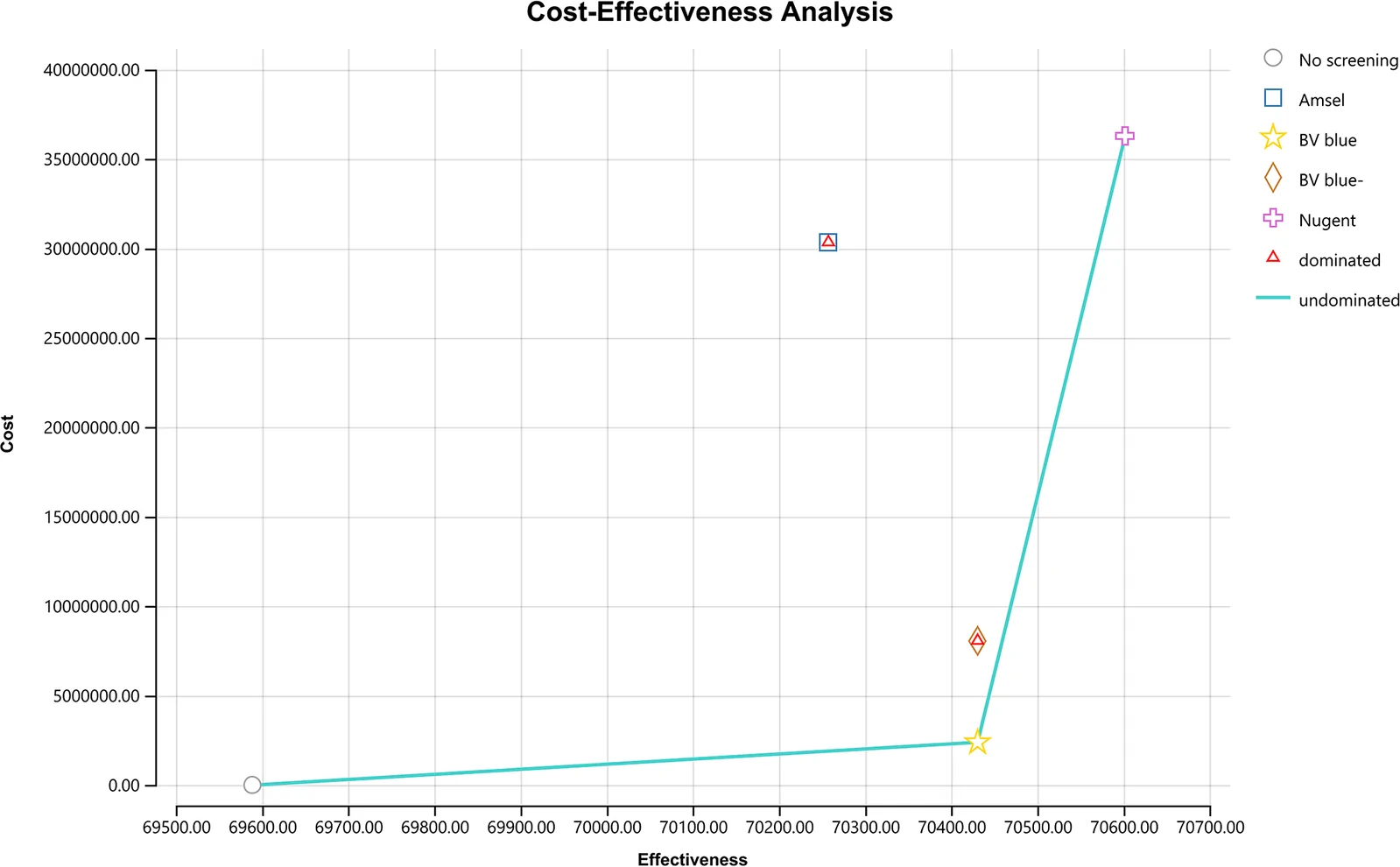
Cost-effectiveness analysis plot

Compared with no screening, BV Blue testing incurred an incremental cost of 2,379,000 CNY and resulted in 842 additional pregnancies, corresponding to an ICER of 2,827 CNY per additional pregnancy, well below the WTP threshold of 99,665 CNY. The sequential BV Blue–Nugent strategy was absolutely dominated by BV Blue testing, with higher costs but no additional pregnancies, and was therefore excluded. The Amsel strategy was likewise absolutely dominated by BV Blue testing, resulting in 173 fewer pregnancies at a higher cost, and was also excluded. Compared with BV Blue testing, Nugent scoring incurred an incremental cost of 33,894,985 CNY and resulted in 171 additional pregnancies, yielding an ICER of 198,051 CNY per additional pregnancy. This value fell below the WTP threshold of 298,995 CNY, meeting the cost-effectiveness criterion. The cost-effectiveness plane is presented in Fig. 2.

### One-way sensitivity analysis

**Fig. 3:**
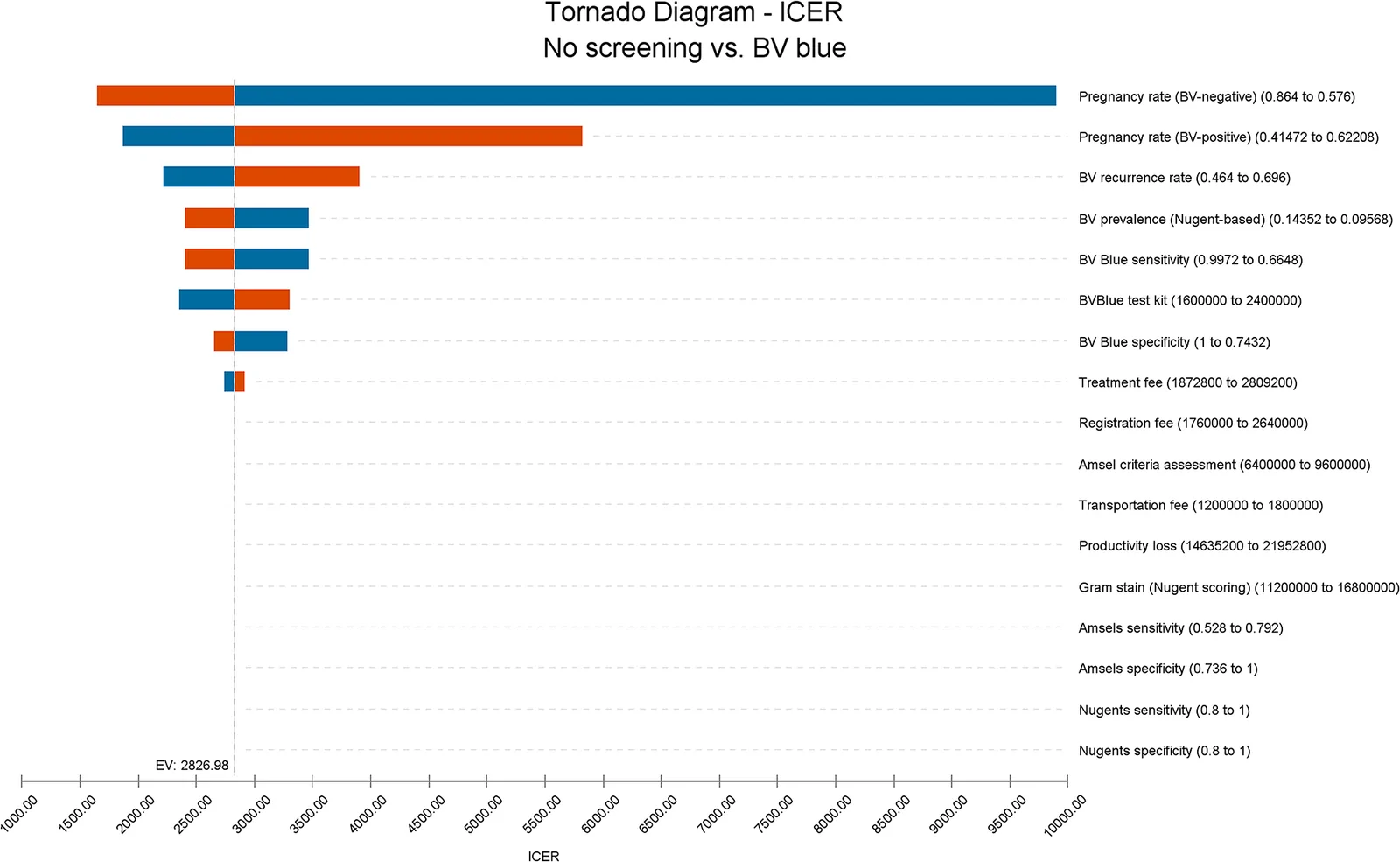
One-Way sensitivity analysis: No screening vs. BV blue

In the comparison between no screening and BV Blue testing (Fig. 3), pregnancy rates among BV-negative and BV-positive women were the most influential parameters, followed by the BV recurrence rate and BV prevalence. Since the ICER remained well below the WTP threshold across all ± 20% parameter variations, the conclusion that BV Blue testing is cost-effective compared with no screening was robust to parameter uncertainty.

**Fig. 4:**
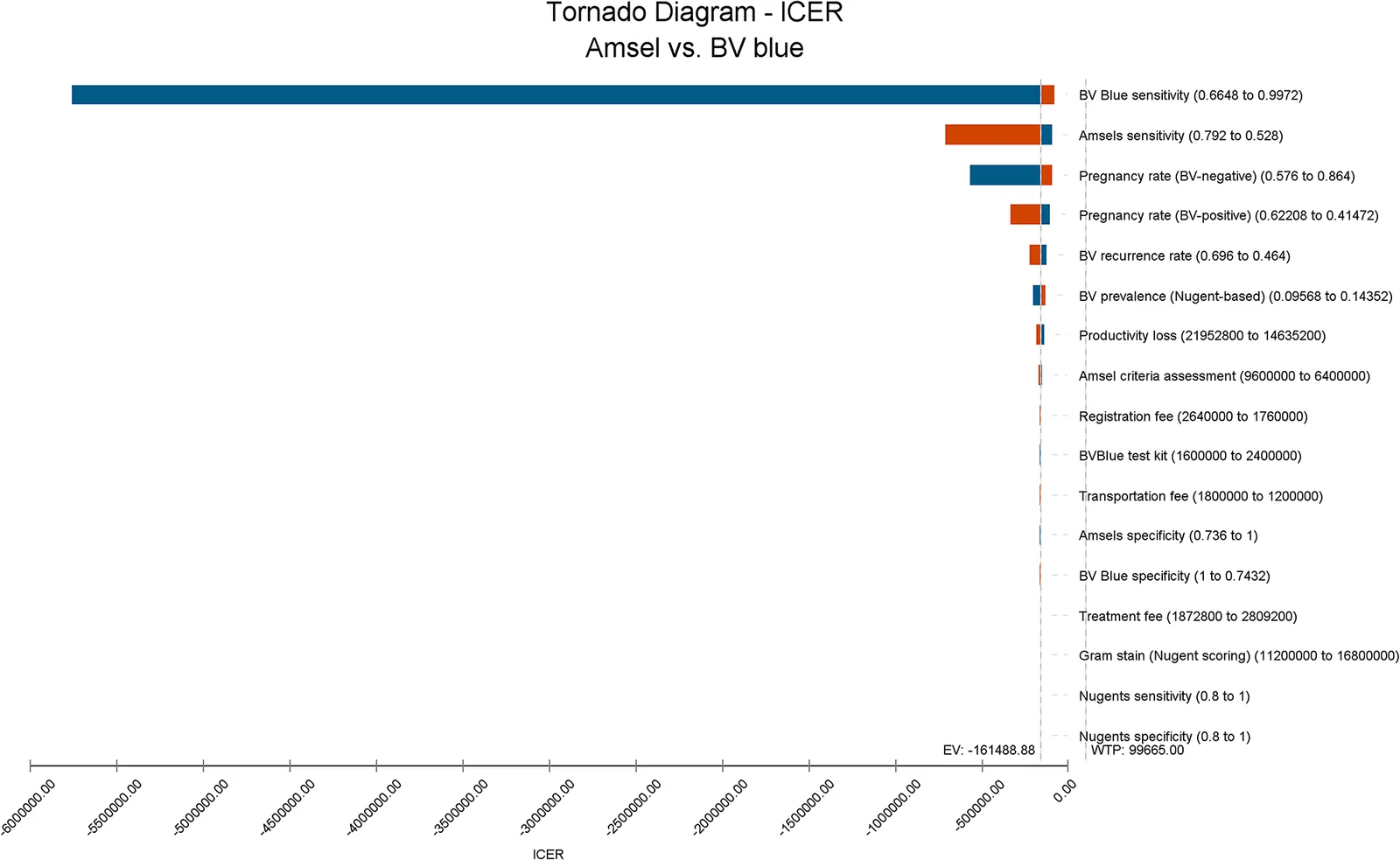
One-Way sensitivity analysis: Amsel vs. BV blue

In the comparison between the Amsel criteria and BV Blue testing (Fig. 4), BV Blue and Amsel sensitivities were the most influential parameters. However, the ICER remained negative across all ± 20% parameter variations, indicating that the absolute dominance of BV Blue testing over the Amsel strategy was robust to parameter uncertainty.

**Fig. 5:**
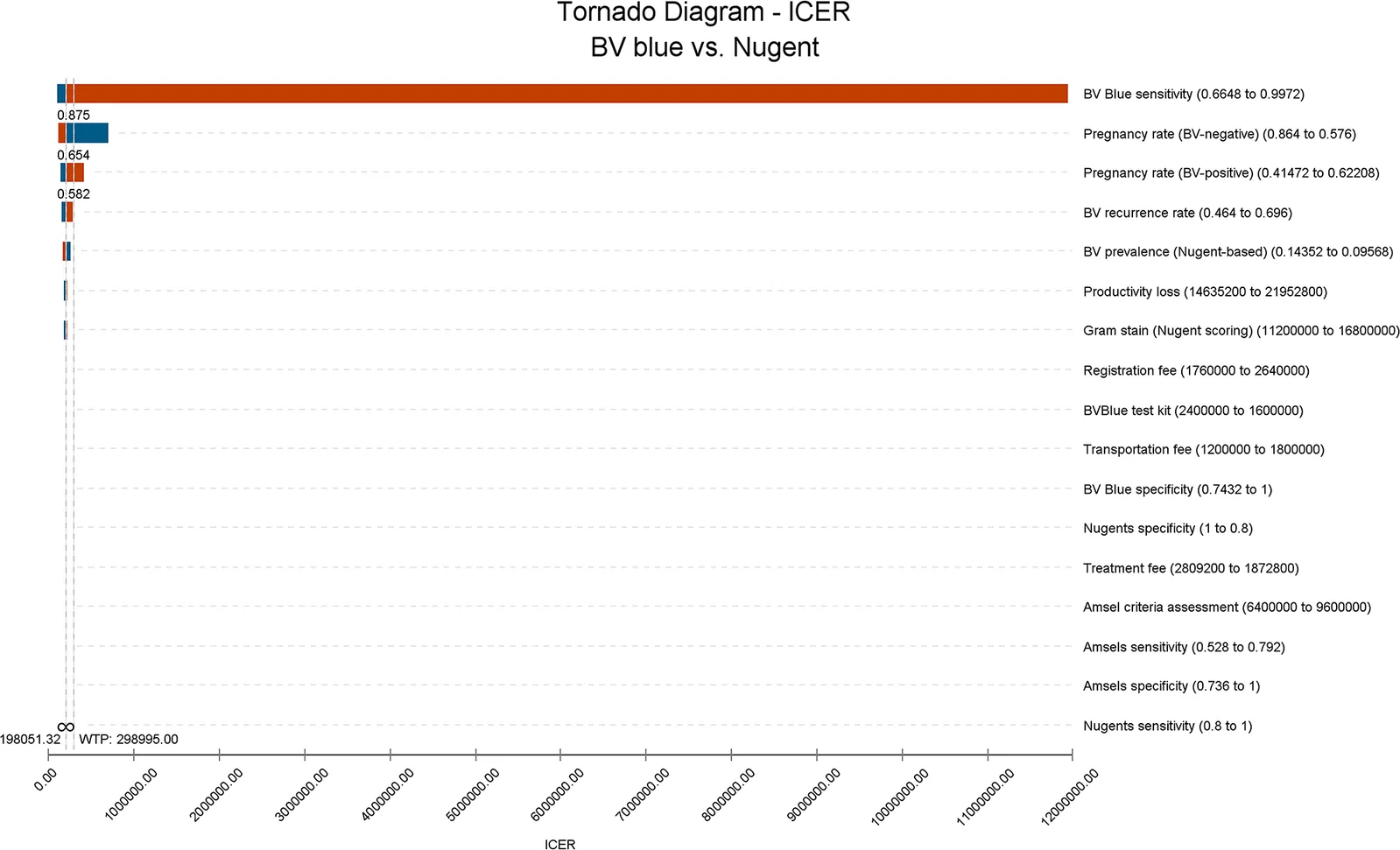
One-Way sensitivity analysis: BV blue vs. Nugent

In the comparison between BV Blue testing and the Nugent scoring (Fig. 5), BV Blue sensitivity was the primary driver of ICER variability, followed by pregnancy rates among BV-negative and BV-positive women. The ICER exceeded the WTP threshold of 298,995 CNY when BV Blue sensitivity > 0.875, the BV-negative pregnancy rate < 0.654, or the BV-positive pregnancy rate > 0.582. This identifies these three parameters as the key determinants of the cost-effectiveness of Nugent scoring relative to BV Blue testing.

### Probabilistic sensitivity analysis

**Fig. 6:**
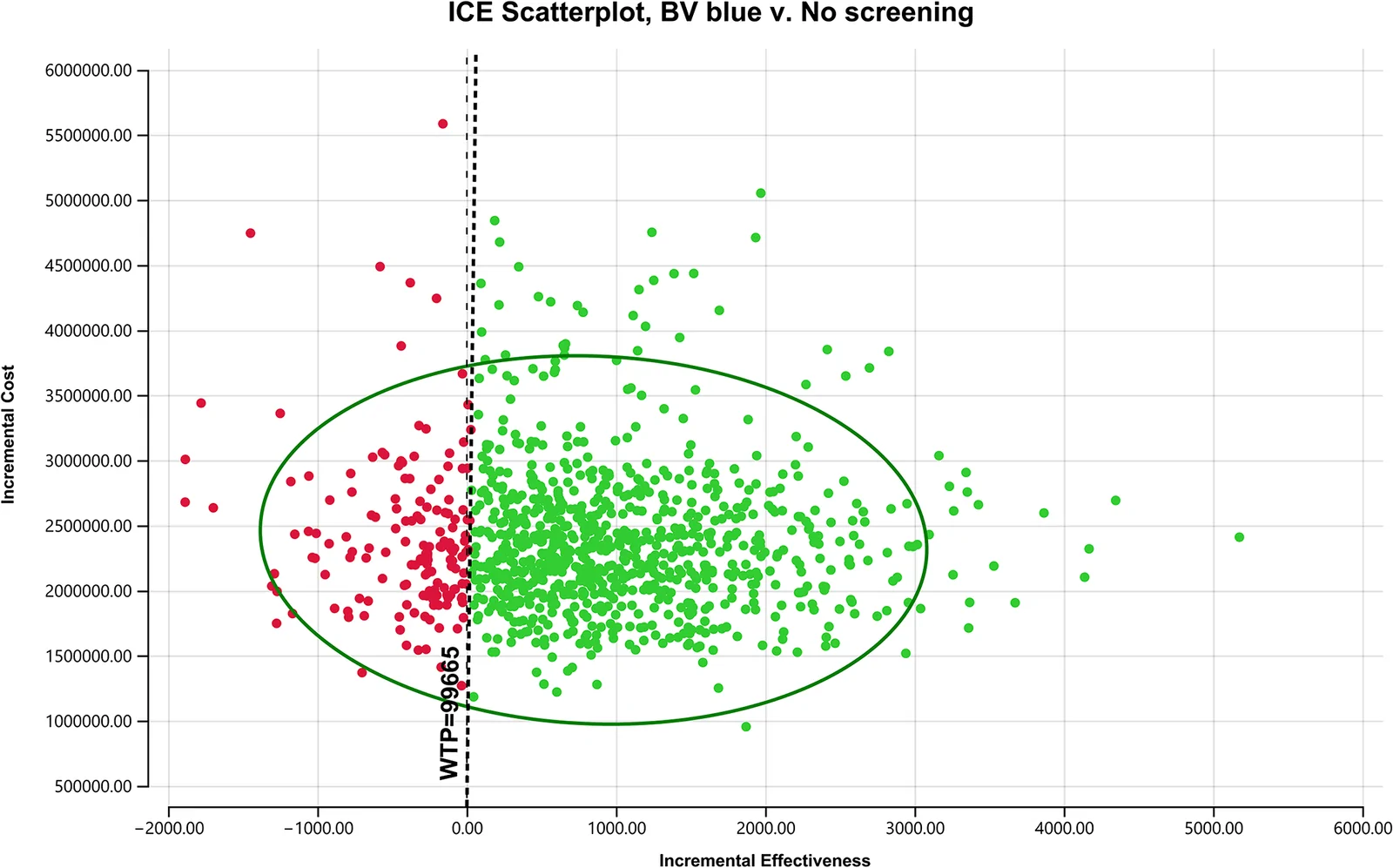
Cost-effectiveness scatter plot: BV blue vs. No screening

**Fig. 7:**
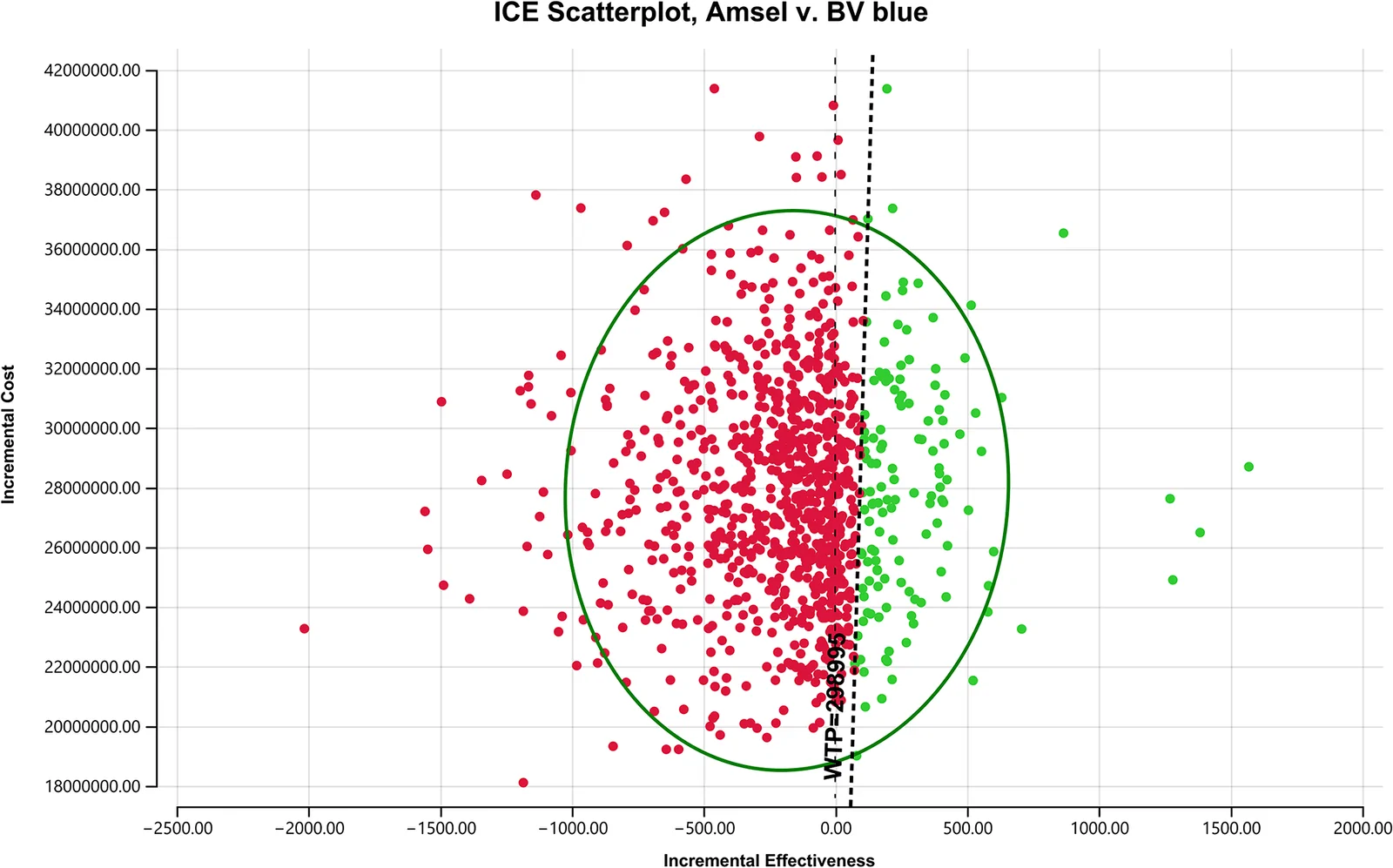
Cost-effectiveness scatter plot: BV blue vs. Amsel

As shown in Fig. 6, in the comparison between BV Blue and no screening, 86.61% of simulation points fell within the C2 quadrant (higher cost, greater effectiveness, ICER < WTP), indicating that BV Blue testing was cost-effective relative to no screening in the majority of simulations. As shown in Fig. 7, in the comparison between the Amsel strategy and BV Blue, 72.66% of simulation points fell within the C6 quadrant (higher cost, lower effectiveness), confirming that BV Blue testing remained the dominant strategy across most simulations.

**Fig. 8:**
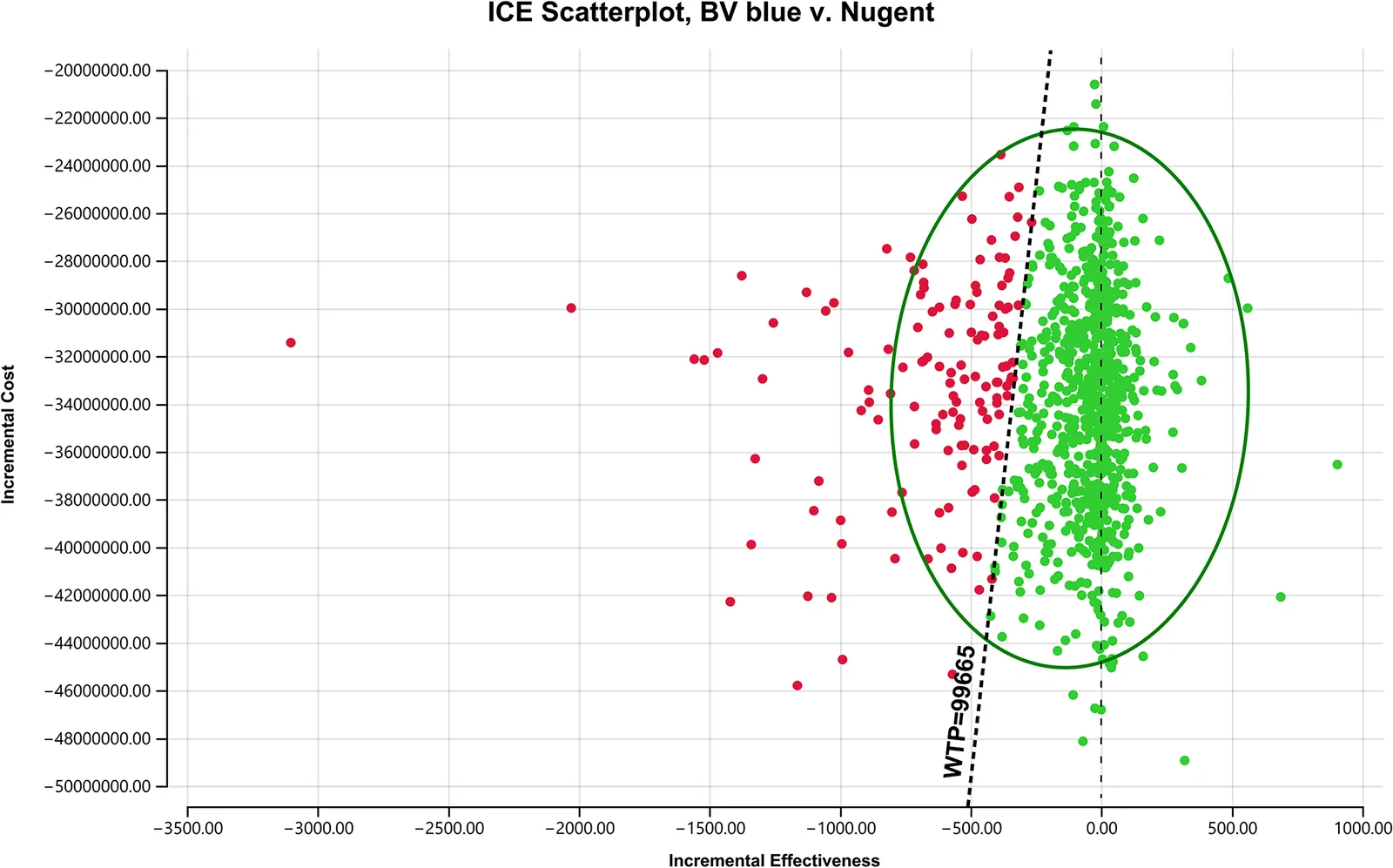
Cost-effectiveness scatter plot: BV blue vs. Nugent

In the comparison between BV Blue and Nugent (Fig. 8), simulated iterations were approximately evenly distributed across three quadrants: 33.16% fell in the C2 quadrant (higher cost, greater effectiveness, ICER < WTP), 32.62% in the C4 quadrant (higher cost, greater effectiveness, ICER > WTP), and 34.22% in the C6 quadrant (higher cost, lower effectiveness), indicating substantial uncertainty in the relative cost-effectiveness of the two strategies. This finding was consistent with the results of the one-way sensitivity analysis. The detailed quadrant distribution is provided in Appendix E of the Supplementary Material.

**Fig. 9:**
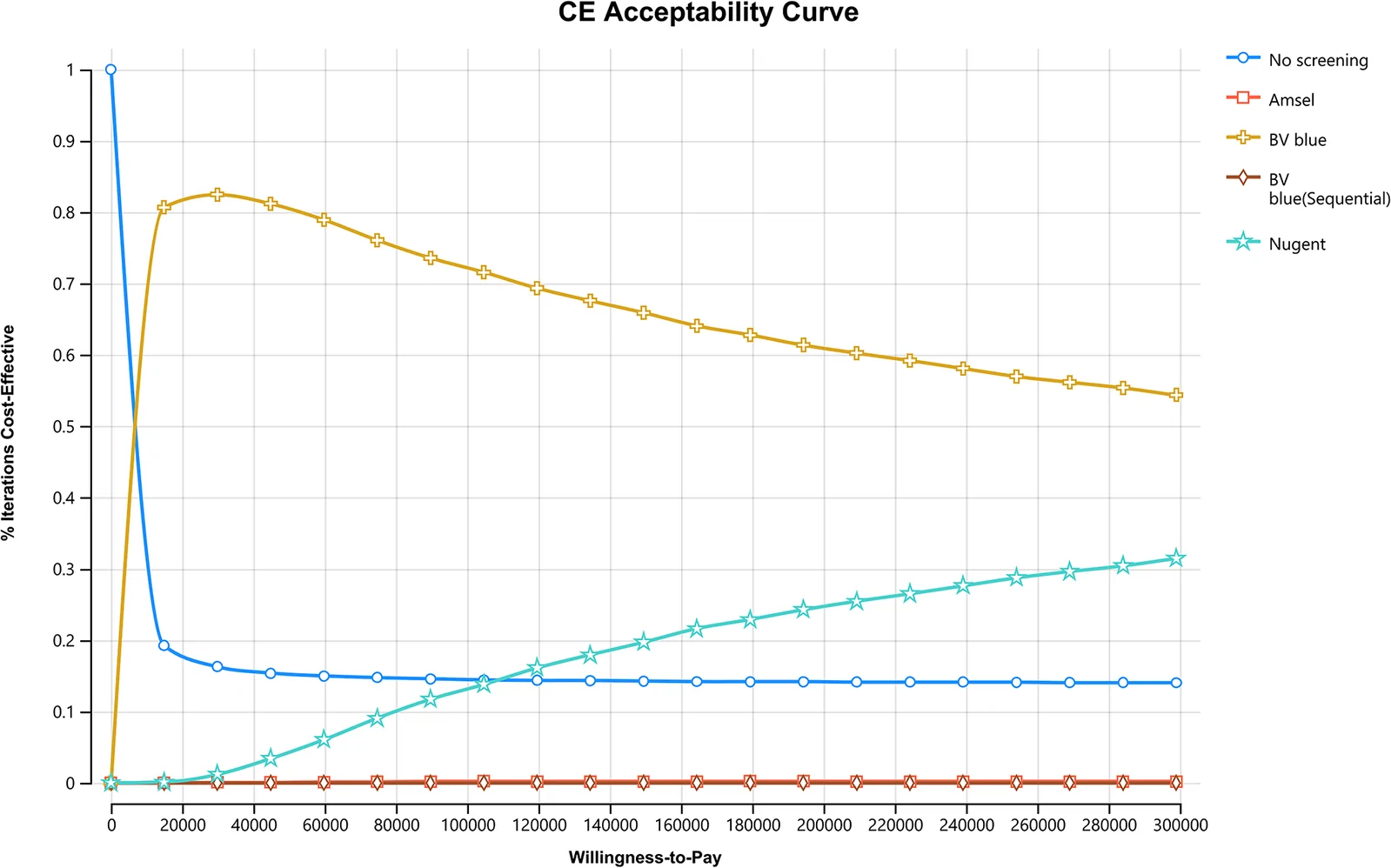
Cost-effectiveness acceptability curve

As shown in Fig. 9, the cost-effectiveness acceptability curve demonstrated that BV Blue consistently had the highest probability of being the optimal strategy across the full range of WTP values. The probability of BV Blue being cost-effective peaked at 83.5% at a WTP of approximately CNY 29,899, then declined gradually as WTP increased, while the probability for Nugent increased steadily with rising WTP values. At the three-fold GDP per capita threshold of CNY 298,995, the cost-effectiveness probabilities of BV Blue and Nugent were 53.98% and 32.06%, respectively. The probability of no screening was highest at low WTP values, declined rapidly, and stabilized at approximately 14% beyond a WTP of CNY 119,467. Amsel and the sequential BV Blue–Nugent strategy had negligible cost-effectiveness probabilities throughout the entire WTP range, consistent with their classification as absolutely dominated strategies in the deterministic analysis. Taken together, these results suggest that BV Blue remains the optimal strategy from a probabilistic perspective at the current WTP threshold.

## Discussion

In current preconception care practice in China, limited accessibility to clinic-based BV screening, low voluntary participation rates, and the limited diagnostic sensitivity of the Amsel criteria may leave a substantial proportion of BV-positive women undetected before conception. We constructed a decision tree model to compare the cost and effectiveness of four BV screening strategies against no screening in a simulated cohort of 100,000 women planning natural conception.

Our findings suggest that incorporating BV Blue rapid testing into China’s national preconception health examination programme represents the most cost-effective approach to improving pregnancy outcomes among all modelled strategies. Wider deployment of BV Blue could meaningfully expand case detection and increase the proportion of women receiving timely treatment. This is broadly consistent with evidence from other settings: Jiao et al. [28] demonstrated that a hypothetical clinician-administered POC test for BV was cost-effective among symptomatic women in the United States, while Saweri et al. [29] found that POC testing and treatment for genital infections during pregnancy was generally cost-effective compared with no screening in low- and middle-income countries, suggesting that the economic advantage of point-of-care approaches may generalise across different clinical contexts and outcome frameworks.

Nugent scoring met the cost-effectiveness criterion under the three-fold GDP per capita threshold (ICER = CNY 198,051 per additional pregnancy), yet the economic case for its routine adoption warrants careful interpretation. No China-specific WTP threshold exists for pregnancies gained as a health outcome. For context, the median total economic burden of IVF-ET — the most commonly used ART procedure in China — was CNY 60,010 per cycle in 2023 [30], with a clinical pregnancy rate of approximately 50% meaning couples typically require multiple cycles. The Nugent ICER is thus approximately 3.3 times the per-cycle IVF-ET burden, suggesting it is not negligible in the Chinese healthcare context.

Sensitivity analyses confirmed the robustness of findings that all screening strategies outperformed no screening, with BV Blue dominating both the sequential and Amsel strategies. However, Nugent’s cost-effectiveness relative to BV Blue was not robust, depending critically on BV Blue sensitivity: at low sensitivity, Nugent added value by detecting missed cases; at high sensitivity, its incremental benefit disappeared and no longer met the threshold.

Cost-effectiveness analysis found that introducing subsequent confirmation steps for all BV Blue-positive populations, although aiming to avoid potential risks of overtreatment, did not significantly improve pregnancy outcomes and led to a substantial increase in medical costs. Perhaps in an environment with limited medical resources or a heavy disease burden, Nugent scoring alone, rather than the sequential strategy, may be considered for screening to strengthen BV management.

Notably, BV Blue-positive results in samples classified as negative by Amsel or Nugent criteria may reflect an underlying dysbiosis dominated by non-Gardnerella anaerobes, such as Prevotella spp., Bacteroides spp., or Mobiluncus spp. [17]. These microorganisms are documented producers of sialidase, a key virulence factor [31, 32], and may remain susceptible to standard metronidazole therapy [33]. Consequently, treating these cases may confer unintended health benefits.

This study has several limitations. First, the decision tree model adopts a one-year time horizon to simulate a single conception cycle; a Markov model would be better suited to capturing the long-term dynamics of a continuous screening programme [34], and future studies should consider extending the time horizon to reflect repeated screening cycles and cumulative reproductive outcomes. Second, real-world adherence to treatment for recurrent BV varies considerably across individuals, making post-recurrence healthcare costs and pregnancy outcomes difficult to estimate with precision; the conservative modelling assumptions adopted here may therefore introduce some uncertainty into parameter estimates [35], and prospective cohort data on treatment adherence and recurrence management would help refine future models. Third, some cost parameters were derived from Nanjing, which may limit the applicability of the findings to other regions, multi-site cost data collection across different healthcare settings in China would strengthen the generalisability of subsequent analyses.

Fourth, the model did not account for implementation-level factors such as test kit availability and women’s willingness to engage in home-based self-testing, both of which could substantially influence real-world programme uptake; future implementation research should incorporate these factors to better inform policy translation. Fifth, pregnancy represents only the initial outcome in the reproductive continuum, and subsequent pregnancy loss carries considerable clinical relevance; as evidence on BV-related health utility values and the causal pathway between BV and miscarriage matures in Chinese populations [36], future modelling efforts should incorporate miscarriage endpoints and quality-adjusted life years to provide a more comprehensive assessment of the health burden. Sixth, ethical constraints preclude randomised controlled trials in preconception BV screening, and the evidence base therefore relies on observational data; well-designed prospective studies and pragmatic trials comparing screening strategies in real-world preconception care settings would be valuable in validating these findings.

## Conclusion

In the preconception setting, all four active screening strategies yielded more pregnancies than no screening, supporting the clinical value of preconception BV detection and treatment. Among them, BV Blue testing and Nugent scoring met the cost-effectiveness criterion, whereas the Amsel-based and sequential BV Blue–Nugent strategies were absolutely dominated and therefore not cost-effective. Probabilistic sensitivity analysis further confirmed that BV Blue testing consistently had the highest probability of being the optimal strategy across the full range of willingness-to-pay values. BV Blue testing therefore represents a promising and robust screening tool for preconception BV management, particularly in primary care settings and resource-limited areas, while Nugent scoring may serve as an alternative where laboratory capacity permits, although its cost-effectiveness relative to BV Blue is sensitive to BV Blue’s diagnostic performance. These findings may provide evidence-based guidance for diagnostic pathway selection within China’s national preconception screening programme and support the ongoing optimisation of preconception BV management strategies.

## Supplementary Information

Below is the link to the electronic supplementary material.

Supplementary Material 1

## Data Availability

No datasets were generated or analysed during the current study.
