# Supplementary material for: Cost-effectiveness analysis of bacterial vaginosis screening strategies in women attempting for natural conception
Source: Cost Eff Resour Alloc. 2026 Jul 15;24:125. doi: 10.1186/s12962-026-00798-x (PMC13651033; doi:10.1186/s12962-026-00798-x)
Supplement: Supplementary file 1 [file 12962_2026_798_MOESM1_ESM.docx]

**Appendix A: Calculation of pregnancy rates for BV-positive and BV-negative women**

- The BV prevalence used in this calculation was 12.91%, derived from a meta-analysis of five large-scale population surveys conducted in China (Appendix B).
- The overall one-year pregnancy rate of 69.4% was derived from a prospective cohort study[1] previously published by our research group. Let X and Y denote the pregnancy rates of BV-positive and BV-negative women of reproductive age, respectively. The following two equations were established:

(1) 12.91% × X + (1 − 12.91%) × Y = 69.4%

This equation states that the weighted average of the pregnancy rates of BV-positive and BV-negative women, weighted by BV prevalence, equals the overall population pregnancy rate of 69.4%.

(2) (1 − X) / (1 − Y) = 1.72

This equation applies the odds ratio of 1.72 for the association between BV and failure to conceive, such that the ratio of the infertility rate among BV-positive women to that among BV-negative women equals 1.72.

- Solving these two equations simultaneously yields pregnancy rates of 51.84% for BV-positive women and 72.00% for BV-negative women.

**Appendix B: Prevalence of bacterial vaginosis among reproductive-aged women in China: A meta-analysis**

Table 1. Characteristics of studies included in the meta-analysis of BV prevalence

| Author | Year | Country | Number of BV patients | Total |
| --- | --- | --- | --- | --- |
| Xu CY | 2011 | China | 553 | 6339 |
| Li XD | 2014 | China | 6391 | 53286 |
| Zhao T | 2022 | China | 164 | 770 |
| Zhang XJ | 2009 | China | 6391 | 53652 |
| Li XD | 2009 | China | 124 | 793 |

Figure 1. Forest plot of BV prevalence in Chinese women of reproductive age

**Appendix C: Sensitivity and specificity of BV Blue: A meta-analysis of diagnostic accuracy**

Table 2. Characteristics of studies included in the diagnostic accuracy meta-analysis of BV Blue

| Author | Year | Country | TP | FN | FP | TN | Total |
| --- | --- | --- | --- | --- | --- | --- | --- |
| Lokken EM | 2022 | Africa | 199 | 44 | 35 | 423 | 701 |
| Myziuk L | 2003 | Unknow | 7 | 1 | 5 | 44 | 57 |
| Bradshaw CS | 2005 | Australia | 108 | 15 | 6 | 119 | 252 |
| Foessleitner P | 2021 | Australia | 81 | 19 | 0 | 100 | 200 |

Figure 2. Forest plot of the sensitivity and specificity of BV Blue

**Appendix D. Notes on Cost Parameter Estimation**

Registration fee: Outpatient registration fees at public hospitals in Nanjing are tiered by physician seniority: general practitioners (12 CNY), associate chief physicians (22 CNY), and chief physicians (35 CNY). As preconception screening clinics are predominantly staffed by associate chief physicians, 22 CNY was used as the base value, with a sensitivity range of 12–35 CNY.

Vaginal discharge examination fee(Amsel): Based on clinical investigation at Nanjing Maternity and Child Health Hospital (60 CNY) and Zhongda Hospital (101 CNY), the mean value of 80 CNY was used as the base value, with a sensitivity range of 60–101 CNY.

Nugent examination fee: Sourced from Nanjing healthcare institution pricing schedules, with 140 CNY as the base value and ±20% (112–168 CNY) as the sensitivity range.

BVBlue test kit: Priced at 20 CNY based on commercial platform data, with ±20% (16–24 CNY) as the sensitivity range.

Metronidazole: A 0.75% metronidazole gel was used; the clinical dispensing unit is one box (7 applicators) at 23.41 CNY, with ±20% as the sensitivity range.

Transportation costs: Estimated at 15 CNY per round trip based on prevailing public transit pricing in Nanjing, with a sensitivity range of 10–50 CNY to account for variation in travel distance and mode of transport.

Productivity losses: Calculated using the human capital approach, based on the mean of the 2024 average annual wages for employees in urban non-private units (160,374 CNY) and private units (83,388 CNY) in Nanjing, as reported by the Nanjing Municipal Bureau of Statistics, converted to an hourly rate assuming 250 working days and 8 hours per day. Each clinic visit was estimated at 3 hours, yielding a base productivity loss of 182.84 CNY per visit, with a sensitivity range of 125.08–240.60 CNY.

**Appendix E. Detailed Quadrant Distribution of ICE Scatterplot Results**

1. Definition of ICE Scatterplot Components

The incremental cost-effectiveness (ICE) scatterplot presents incremental effectiveness (IE) on the x-axis and incremental cost (IC) on the y-axis. A willingness-to-pay (WTP) threshold line passing through the origin — with a slope of 99,665 CNY for comparisons involving no screening, Amsel, or BV Blue testing as the comparator at the 1× GDP threshold, or 298,995 CNY for the BV Blue vs. Nugent comparison at the 3× GDP threshold — divides the plane into six regions as follows:

**C1 (Quadrant IV):** IE > 0 and IC < 0. The new strategy is more effective and less costly — dominant. No ICER calculation required.

**C2 (Quadrant I, below WTP line):** IE > 0 and IC > 0, with ICER below the WTP threshold. The new strategy is more effective at an acceptable cost — cost-effective.

**C4 (Quadrant I, above WTP line):** IE > 0 and IC > 0, with ICER exceeding the WTP threshold. The new strategy is more effective but too costly — not cost-effective.

**C6 (Quadrant II):** IE < 0 and IC > 0. The new strategy is less effective and more costly — dominated.

**C3 (Quadrant III, above WTP line):** IE < 0 and IC < 0, with ICER below the WTP threshold. The cost savings are proportionally large relative to the effectiveness loss — cost-effective.

**C5 (Quadrant III, below WTP line):** IE < 0 and IC < 0, with ICER exceeding the WTP threshold. The cost savings are insufficient to offset the effectiveness loss — not cost-effective.

Table 3. BV Blue Testing vs. No-Screening Strategy (1,000 Monte Carlo Simulations)

| **Component** | **Quadrant** | **Incremental Effectiveness (IE)** | **Incremental Cost (IC)** | **ICER Criterion** | **Economic Interpretation** | **Frequency** | **Proportion** |
| --- | --- | --- | --- | --- | --- | --- | --- |
| C1 | Quadrant IV | IE > 0 | IC < 0 | Dominant | More effective and less costly | 0 | 0.00% |
| **C2** | Quadrant I (below WTP line) | IE > 0 | IC > 0 | **ICER < 99,665 CNY** | **Cost-effective** | 8,661 | **86.61%** |
| C3 | Quadrant III (above WTP line) | IE < 0 | IC < 0 | ICER < 99,665 CNY | Cost-effective (cost-saving) | 0 | 0.00% |
| C4 | Quadrant I (above WTP line) | IE > 0 | IC > 0 | ICER > 99,665 CNY | Not cost-effective | 24 | 0.24% |
| C5 | Quadrant III (below WTP line) | IE < 0 | IC < 0 | ICER > 99,665 CNY | Not cost-effective (insufficient saving) | 0 | 0.00% |
| **C6** | Quadrant II | IE < 0 | IC > 0 | **Dominated** | **Less effective and more costly** | 1,315 | **13.15%** |
| Total | — | — | — | — | — | 10,000 | 100% |

Table 4. Amsel Strategy vs. BV Blue Strategy (1,000 Monte Carlo Simulations)

| **Component** | **Quadrant** | **Incremental Effectiveness (IE)** | **Incremental Cost (IC)** | **ICER Criterion** | **Economic Interpretation** | **Frequency** | **Proportion** |
| --- | --- | --- | --- | --- | --- | --- | --- |
| C1 | Quadrant IV | IE > 0 | IC < 0 | Dominant | More effective and less costly | 0 | 0.00% |
| **C2** | Quadrant I (below WTP line) | IE > 0 | IC > 0 | **ICER < 99,665 CNY** | **Cost-effective** | **1,405** | **14.05%** |
| C3 | Quadrant III (above WTP line) | IE < 0 | IC < 0 | ICER < 99,665 CNY | Cost-effective (cost-saving) | 0 | 0.00% |
| C4 | Quadrant I (above WTP line) | IE > 0 | IC > 0 | ICER > 99,665 CNY | Not cost-effective | 1,329 | 13.29% |
| C5 | Quadrant III (below WTP line) | IE < 0 | IC < 0 | ICER > 99,665 CNY | Not cost-effective (insufficient saving) | 0 | 0.00% |
| C6 | Quadrant II | IE < 0 | IC > 0 | **Dominated** | **Less effective and more costly** | 7,266 | 72.66% |
| Total | — | — | — | — | — | 10,000 | 100% |

Table 5. BV Blue Strategy vs. Nugent Strategy (1,000 Monte Carlo Simulations)

| **Component** | **Quadrant** | **Incremental Effectiveness (IE)** | **Incremental Cost (IC)** | **ICER Criterion** | **Economic Interpretation** | **Frequency** | **Proportion** |
| --- | --- | --- | --- | --- | --- | --- | --- |
| C1 | Quadrant IV | IE > 0 | IC < 0 | Dominant | More effective and less costly | 0 | 0.00% |
| **C2** | Quadrant I (below WTP line) | IE > 0 | IC > 0 | **ICER < 298,995 CNY** | **Cost-effective** | **3,316** | **33.16%** |
| C3 | Quadrant III (above WTP line) | IE < 0 | IC < 0 | ICER < 298,995 CNY | Cost-effective (cost-saving) | 0 | 0.00% |
| C4 | Quadrant I (above WTP line) | IE > 0 | IC > 0 | **ICER > 298,995CNY** | **Not cost-effective** | **3,262** | **32.62%** |
| C5 | Quadrant III (below WTP line) | IE < 0 | IC < 0 | ICER > 298,995CNY | Not cost-effective (insufficient saving) | 0 | 0.00% |
| C6 | Quadrant II | IE < 0 | IC > 0 | **Dominated** | **Less effective and more costly** | **3,422** | **34.22%** |
| Total | — | — | — | — | — | 10,000 | 100% |
